# Pancreatic Cancer Presenting to the Dermatology Clinic

**DOI:** 10.7759/cureus.49908

**Published:** 2023-12-04

**Authors:** Sharon Pan, Radhika Shah, Shannon C Brown

**Affiliations:** 1 Dermatology, Baylor Scott & White Medical Center - Temple, Temple, USA

**Keywords:** medical dermatology, general dermatology, pancreatic cancer, dermatology, jaundice, skin of color

## Abstract

In patients with skin of color, jaundice may present more discretely, which can lead to a delay in diagnosing underlying disease and widening racial disparity gaps. It is important for clinicians to recognize the subtleties of jaundice to achieve the most optimal outcomes for patients. Careful examination of the sclera and palms, sites where yellowing is most obvious, as well as asking patients if they have noticed any skin color changes can be beneficial. We present a case of a patient who presented to the dermatology clinic with jaundice and pruritus refractory to standard treatment, ultimately leading to a diagnosis of pancreatic cancer.

## Introduction

Itch is the most common complaint among black patients presenting to the dermatologist [1]. Pruritus disproportionally affects skin of color (SOC) due to epidemiological and pathomechanistic variances such as inequities in care and structural differences in the skin [2]. In addition to an increased prevalence of pruritic conditions such as atopic dermatitis in patients with SOC, increased melanin in the skin may mask subtle changes in skin tone caused by jaundice, leading to a delay in the diagnosis of internal diseases. When examining a patient for jaundice, physicians are taught to examine the sclera for discoloration. However, discoloration of the sclera can be a normal variant in patients with SOC due to increased melanin of the sclera and conjunctiva [3]. Collectively, these variations make it challenging to evaluate a new patient with skin of color for pruritus, widening the racial disparity gap in dermatology. We present a case of a Fitzpatrick VI skin type patient who presented to the dermatology clinic with jaundice and refractory pruritus, ultimately leading to a diagnosis of pancreatic cancer.

## Case presentation

A 66-year-old African American female with no prior dermatologic history presented to the dermatology clinic for a two-week history of severe pruritus on her trunk and upper extremities. She reported an abrupt onset of pruritus and gastrointestinal distress after eating “bad chicken” at a social event. Approximately two weeks after the start of her symptoms, she was prescribed hydroxyzine 10 mg three times a day and received an intramuscular triamcinolone injection by her primary care provider with no relief. An abdominal exam performed at the primary care clinic was unremarkable. Her exam in our clinic revealed xerosis and lichenified plaques on both arms with edematous excoriations, as well as innumerable small, rough, sandpaper-like papules scattered on the posterior arms bilaterally. She was initially suspected to have atopic dermatitis (AD) with dermatographism and subsequently started on cetirizine, hydroxyzine, triamcinolone 0.1% ointment, and pramoxine cream. The papular texture on her posterior arms was suggestive of keratosis pilaris, which further corroborated a likely diagnosis of an atopic dermatitis exacerbation.

**Figure 1:**
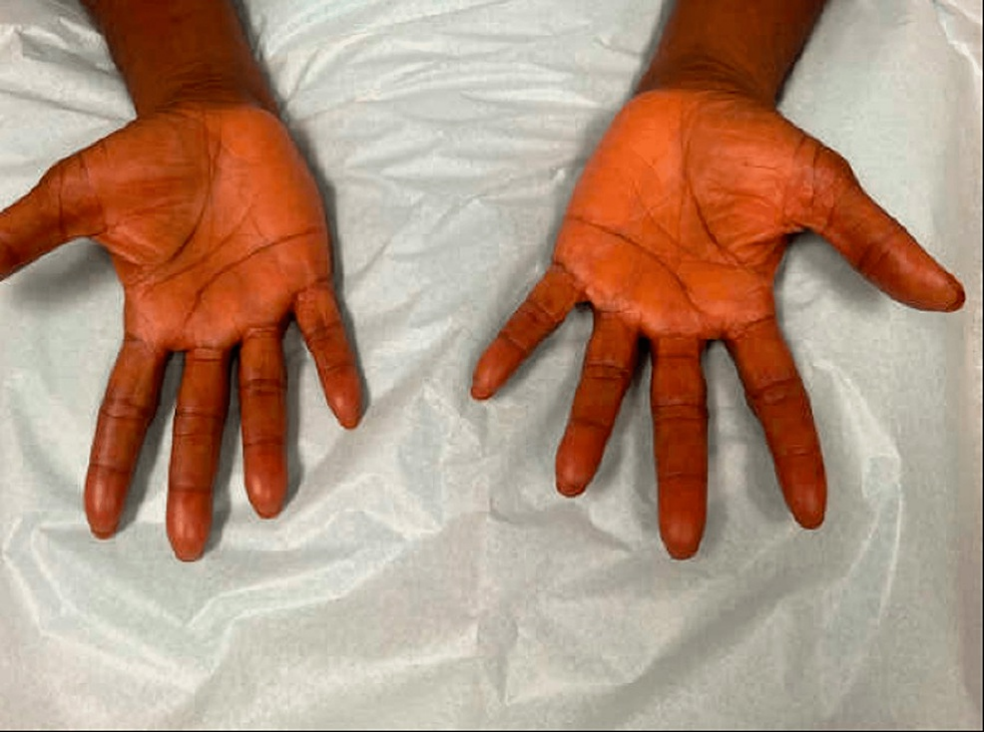
Bilateral palms with background yellowing

**Figure 2:**
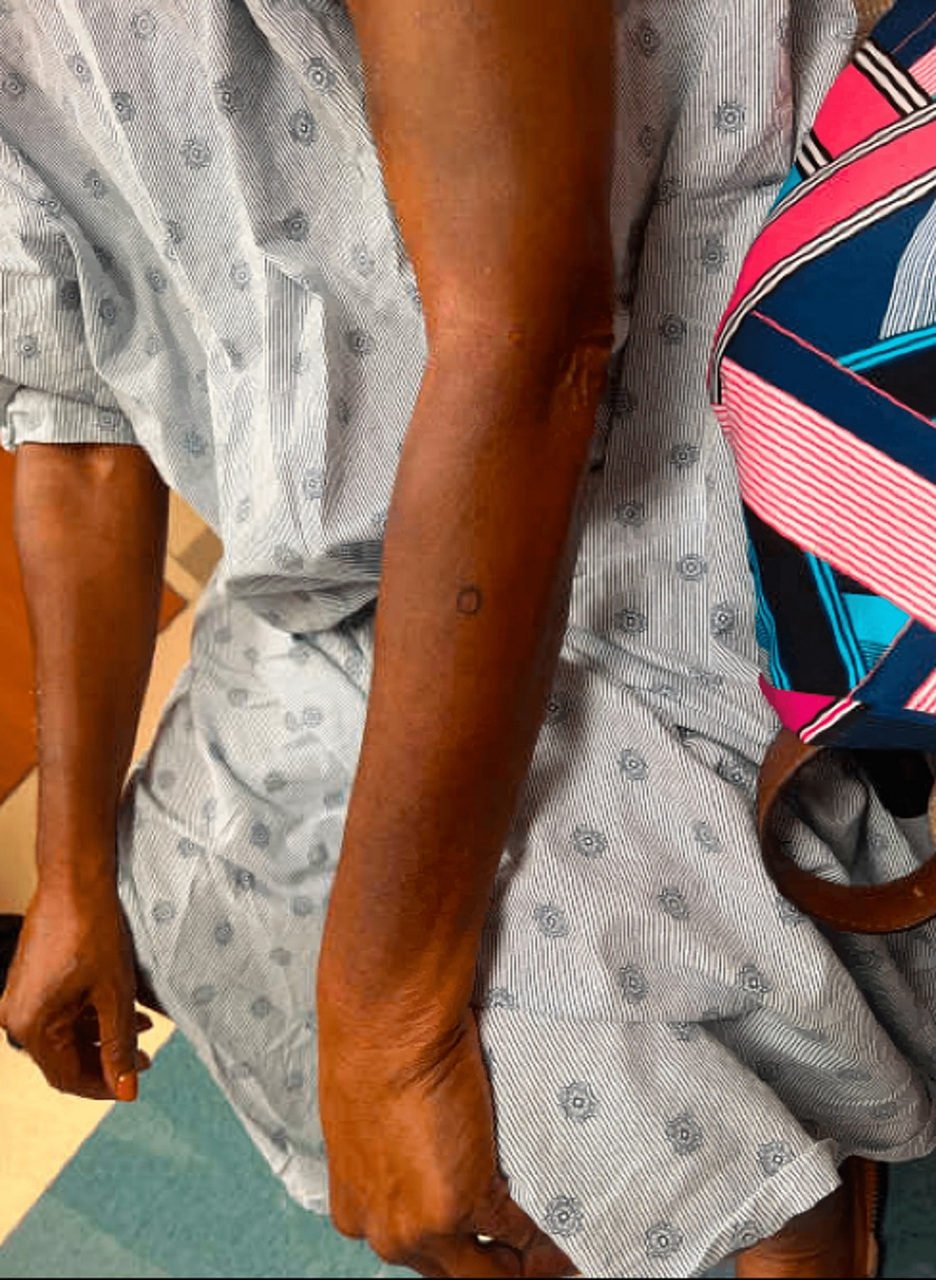
Bilateral forearms with background yellowing

Two weeks later, she returned with complaints of worsening pruritus. She reported feeling unwell, recently having pale, loose stools, decreased appetite, and weight loss. A physical exam showed subtle yellowing of the skin, especially on her palms (Figure 1), distal forearms (Figure 2), and sclera. We obtained a skin biopsy to rule out a primary dermatologic process such as one of eczematous or psorisiform origin and pursued a workup for a gastrointestinal etiology. Labs revealed elevated aspartate aminotransferase (AST), alanine transaminase (ALT), total bilirubin, alkaline phosphatase, lactate dehydrogenase (LDH), and lipase (Table 1). The following day, the lipase increased over two-fold. Abdominal CT evaluation showed a concerning 3.7 x 3.2 cm mass at the pancreatic head with dilation of the main pancreatic duct and the intra/extrahepatic biliary duct, which was diagnosed as pancreatic adenocarcinoma shortly thereafter. The patient then underwent upper endoscopy and endoscopic retrograde cholangiopancreatography (ERCP) with the placement of a metal stent across the malignant biliary stricture. Following these procedures, a chest CT showed no evidence of metastatic disease and improved pancreatic duct dilation. The patient soon established care with oncology, consulted with surgery, and was deemed to have localized, potentially operable pancreatic cancer. She was planned for neoadjuvant chemotherapy with FOLFIRINOX (oxaliplatin, irinotecan, fluorouracil). Given the size and extent of the disease at the time of detection, our patient is potentially eligible for future resection and will undergo repeat CT imaging after a few chemotherapy cycles to assess tumor size for consideration of Whipple surgery.

**Table 1:** Selected initial labs of the patient after presenting to the dermatology clinic AST = aspartate aminotransferase; ALT = alanine transaminase; LDH = lactate dehydrogenase

Selected lab parameters	6/6/23	6/7/23	Reference
Total bilirubin	28.7	25.5	0.2-1.2 mg/dL
Direct bilirubin	22	-	0.0-0.4 mg/dL
Alkaline phosphatase	242	215	34-130 IU/L
AST	215	180	0-40 U/L
ALT	290	245	0-68 U/L
LDH	529	-	84-246 U/L
Lipase	474	1,026	8-78 U/L

## Discussion

Our patient initially presented to their primary care and our dermatology clinic with complaints of pruritus and only later developed noticeable jaundice. When she initially presented to the dermatology clinic, there were no noticeable skin color changes; however, on the second visit, it was apparent she had developed jaundice when compared to her initial presentation. Non-white patients have been noted to have a higher burden of disease associated with chronic pruritus due to epidemiological and pathomechanistic variances [2,4,5]. Structural variances in darker skin include increased trans-epidermal water loss, decreased ceramides, increased size of mast cells, and lower pH of the stratum corneum, leading to an increased prevalence of xerosis and atopic dermatitis, common causes of itch in this population [2,6]. African American patients are also at high risk for having systemic comorbidities associated with pruritus [2]. Thus, there is a need for clinicians to not only be aware of these disparities but also well equipped to work up the patient.

Jaundice can be difficult to appreciate in skin of color, which can delay the diagnosis of diseases such as liver dysfunction and possible malignancy. In the case of pancreatic cancer, symptoms are frequently non-specific, leading to persisting challenges in early detection and slow improvements in survival rates over the past few decades [7]. Our patient’s jaundice was best visualized in her sclera and palms, often where yellowing can best be appreciated in darker skin types. Unfortunately, yellowing of the sclera may be a normal finding with aging in darker skin type populations due to increased pigment in the sclera and conjunctiva, making it challenging to evaluate new patients without a baseline comparison [3]. Asking patients what color their skin/eyes normally are can help detect even the most subtle color changes. A thorough history and review of systems can clue clinicians into an early diagnosis, which is particularly important in the setting of aggressive diseases such as pancreatic cancer [7,8]. In particular, asking about changes in bowel habits, weight, appetite and food intake, and energy level can be helpful, as seen in our patient. Initially, labs may include a complete blood count inclusive of hepatic, renal, and thyroid function panels [2]. If patients have a diffuse itch for less than 12 months, are over 60 years old, are male, or have a history of liver disease and tobacco use, they are at increased risk for having an underlying malignancy [2]. Aside from cholestatic malignancies, other malignancies, such as Hodgkin lymphoma, can cause pruritus without jaundice due to unknown mechanisms. A previous study reported that 18% of patients with gallbladder or biliary malignancies and symptoms of pruritus had documented skin eruptions [9]. For patients with pancreatic or liver cancer and symptoms of pruritus, a little over 20% had documented skin eruptions (21% and 23%, respectively) [9]. These three cancers were among the malignancies with the lowest rate of skin eruptions in the study, which included a little over 40 malignancies, which is consistent with the understanding that the mechanism by which these cancers cause pruritus is through cholestasis, which would not typically cause a visible skin eruption [9]. Our patient had skin findings on exam that were suggestive of consequential skin changes due to the pruritus (“itch that rashes”), rather than the pruritus being secondary to the lesions. Most pancreatic cancers (approximately 70%) are located at the head of the pancreas, with at least half presenting with jaundice and many with complaints of pruritus [10,11]. To our knowledge, there has not yet been a specific discussion accompanied by clinical images of jaundice in patients with SOC.

## Conclusions

Our patient’s case highlights the importance of considering underlying non-dermatologic disease in patients presenting with dermatologic findings refractory to conventional treatment. Although our patient attended an event after which food poisoning may have been a likely culprit for her initial gastrointestinal symptoms, the worsening skin complaints along with the development of systemic symptoms alerted consideration of a possible aggressive underlying disease process. Physicians should remain aware of the variations in which jaundice may present in SOC patients and that worsening, refractory pruritus may warrant investigation of non-dermatologic causes. We present this case to help increase awareness of the subtleties of jaundice in SOC, which may help expedite referral to necessary specialties and improve patient outcomes in the event of underlying disease.
